# Factors influencing the sinus membrane thickness in edentulous regions: a cone-beam computed tomography study

**DOI:** 10.1186/s40729-021-00298-y

**Published:** 2021-03-02

**Authors:** Motohiro Munakata, Kikue Yamaguchi, Daisuke Sato, Naoko Yajima, Noriko Tachikawa

**Affiliations:** 1 Department of Implant Dentistry, Showa University School of Dentistry, 2-1-1, Kita-Senzoku, Ota-ku, 145-8515 Tokyo, Japan; 2 Tokyo Medical and Dental University Dental Hospital, Tokyo Medical and Dental University, Tokyo, Japan

**Keywords:** Sinus augmentation, Sinus mucosal thickness, Nasal septum deviation, Periapical lesions, Time following tooth extraction

## Abstract

**Background:**

During implant treatment in the maxillary molar area, maxillary sinus floor augmentation is often performed to ameliorate the reduced alveolar bone height attributable to bone remodeling and pneumatization-induced expansion of the maxillary sinus. However, this augmentation may cause complications such as misplaced implants, artery damage, and maxillary sinus mucosal perforation; infections like maxillary sinusitis; and postsurgical complications such as bone graft leakage and postoperative nasal hemorrhaging. To reduce the complications during maxillary sinus floor augmentation and postoperative infections, we performed retrospective investigations of various systemic and local factors that influence pre-operative sinus mucosal thickness (SMT) by using cone-beam computed tomography (CBCT). Subjects included patients who underwent maxillary sinus floor augmentation in an edentulous maxillary molar area with a lateral approach. Pre-operative SMT, existing bone mass, and nasal septum deviation were measured using CBCT images. Relationships between SMT and the following influencing factors were investigated: (1) age, (2) sex, (3) systemic disease, (4) smoking, (5) period after tooth extraction, (6) reason for tooth extraction, (7) residual alveolar bone height (RBH), (8) sinus septa, and (9) nasal septum deviation. Correlations were also investigated for age and RBH (*p* < 0.05).

**Results:**

We assessed 35 patients (40 sinuses; 11 male, 24 female). The average patient age was 58.90 ± 9.0 years (males, 57.9 ± 7.7 years; females, 59.9 ± 9.4 years; age range, 41–79 years). The average SMT was 1.09 ± 1.30 mm, incidence of SMT > 2 mm was 25.0%, incidence of SMT < 0.8 mm was 50.0%, and the average RBH was 2.14 ± 1.02 mm. The factors that influenced SMT included sex (*p* = 0.0078), period after tooth extraction (*p* = 0.0075), reason for tooth extraction (*p* = 0.020), sinus septa (*p* = 0.0076), and nasal septum deviation (*p* = 0.038).

**Conclusions:**

Factors associated with higher SMT included male sex, interval following tooth extraction < 6 months, periapical lesions, sinus septa, and nasal septum deviation. Factors associated with SMT > 2 mm were sex and reason for tooth extraction, while factors associated with SMT < 0.8 mm were time following tooth extraction and nasal septum deviation. Despite the limitations of this study, these preoperative evaluations may be of utmost importance for safely conducting maxillary sinus floor augmentation.

## Background

Maxillary sinus floor augmentation is often required during implant treatment in the maxillary molar area because bone remodeling from loss of teeth and expansion of the maxillary sinus over time due to pneumatization result in insufficient alveolar bone height. Reports on changes in the maxillary sinus due to loss of teeth have indicated significant expansion of the maxillary sinus (1–5 mm) following tooth extraction when the tooth root was either protruding into the maxillary sinus or when it elevated the maxillary sinus floor [1]. Furthermore, reports have indicated an extremely close relationship between the maxillary sinus floor and the tooth/alveolar bone, with the distance between the root of the tooth and the maxillary sinus decreasing with age or with tooth loss [2]. Maxillary sinus floor augmentation has been established as a highly predictive treatment method during implant treatments in the maxillary molar area. Systematic reviews have indicated high survival rates of implants that underwent maxillary sinus floor augmentation, with 3-year and 5-year survival rates of 90.1% and 92%–100%, respectively [3, 4].

However, maxillary sinus floor augmentation has also often been reported to cause complications during surgery, such as misplaced implants, artery damage, and maxillary sinus mucosal perforation; infections like maxillary sinusitis; and postoperative complications such as bone graft leakage and nasal hemorrhage [5–7]. Sinus mucosal perforation was the most frequent complication during surgery, with an incidence of 0–58.3%, whereas maxillary sinusitis was a frequent postoperative complication, with an incidence of 3–20%, and both of these complications were related to a medical history of chronic sinusitis, smoking, and sinus septa, as well as stenosis of the ostiomeatal complex (OMC) due to a deviated nasal septum or concha bullosa [7].

Increased sinus mucosal thickness (SMT) could be caused by a variety of inflammatory symptoms, and the influencing factors include patient-related factors like age or smoking history; tooth-related factors such as the presence of periapical lesions, severity of periodontal diseases, and the extent of alveolar bone loss; and factors related to the morphology of the maxillary sinus, such as the measurement section and the sinus septa [8–10]. Reports have indicated ostium obstruction in 59.3% of patients with SMT > 5 mm [11]. Cagici et al., Janner et al., and Shanbhag et al. reported that the anatomy of the paranasal cavity influenced cases where the SMT > 2 mm, and that the mucosa could be seen only at a thickness of 2 mm or above; therefore, 2 mm was historically considered a reliable threshold for pathological mucosal swelling [12–14]. Reports have also indicated that membrane thickness was correlated with the sinus mucosal perforation rate, and that sinus mucosal perforation risk increased when SMT was < 0.8 mm or > 2 mm, showing that SMT influenced maxillary sinus floor augmentation procedures such as sinus mucosal perforation and increases in post-operative inflammation [15, 16].

However, existing research on the SMT has predominantly been conducted under conditions where teeth were present, and very few studies have investigated the relationship between SMT and OMC in edentulous areas or assessed the factors related to the reasons for tooth extraction and the time after tooth extraction.

Therefore, the present study used cone-beam computed tomography (CBCT) to investigate various factors that influence preoperative SMT (sex, age, systemic diseases, time after tooth extraction, smoking, reason for tooth extraction, residual bone height, sinus septa, nasal septum deviation) with the aim of reducing complications during maxillary sinus floor augmentation and the onset of postoperative infection.

## Methods

### Subjects

The subjects were patients who underwent maxillary sinus floor augmentation in an edentulous maxillary molar area with a lateral approach at the Department of Implant Dentistry at Showa University Dental Hospital from June 2018 to June 2020.

The inclusion criteria were (1) presence of a partial edentulation in the posterior region of the maxilla; (2) residual bone height of 5 mm or less; (3) at least one CBCT scan prior to a maxillary sinus floor augmentation procedure. Exclusion criteria were as follows: (1) preoperative SMT over 5 mm as determined by CBCT images, (2) presence of non-transparent objects in the maxillary sinus, such as mucinous cysts or polyps, (3) a medical history of paranasal sinus diseases, (4) respiratory diseases such as bronchitis or bronchial asthma, (5) uncontrolled diabetes, (6) CBCT imaging within 3 months of tooth extraction, (7) age under 40 years, and (8) unclear reasons for tooth extraction in the edentulous area.

### Methods

The study protocol was conducted in full accordance with the ethical principles established in the World Medical Association Declaration of Helsinki of 1975 as revised in 2000, and approved by the Ethical Committee of the Showa University Dental Hospital (Approval Number: DH2019-046).

### CBCT image analysis and assessment

#### Measurements to determine the SMT and residual alveolar bone height (RBH) (Fig. 1)

A wax-up was conducted using a study model and a barium-incorporated resin was used to create a surgical template (KaVo 3D exam; KaVo Dental Systems, Biberach). Using the method described by Rapani et al. [17], the bone height and SMT were measured in sagittal computer tomography (CT) images on the line perpendicular to the imaginary occlusal plane from the tooth crown center in each edentulous area. In cases with multiple edentulous areas, the minimum residual alveolar bone height and maximum SMT of each subject were used as the measurement values for the bone height and mucosal thickness, respectively.

**Fig. 1:**
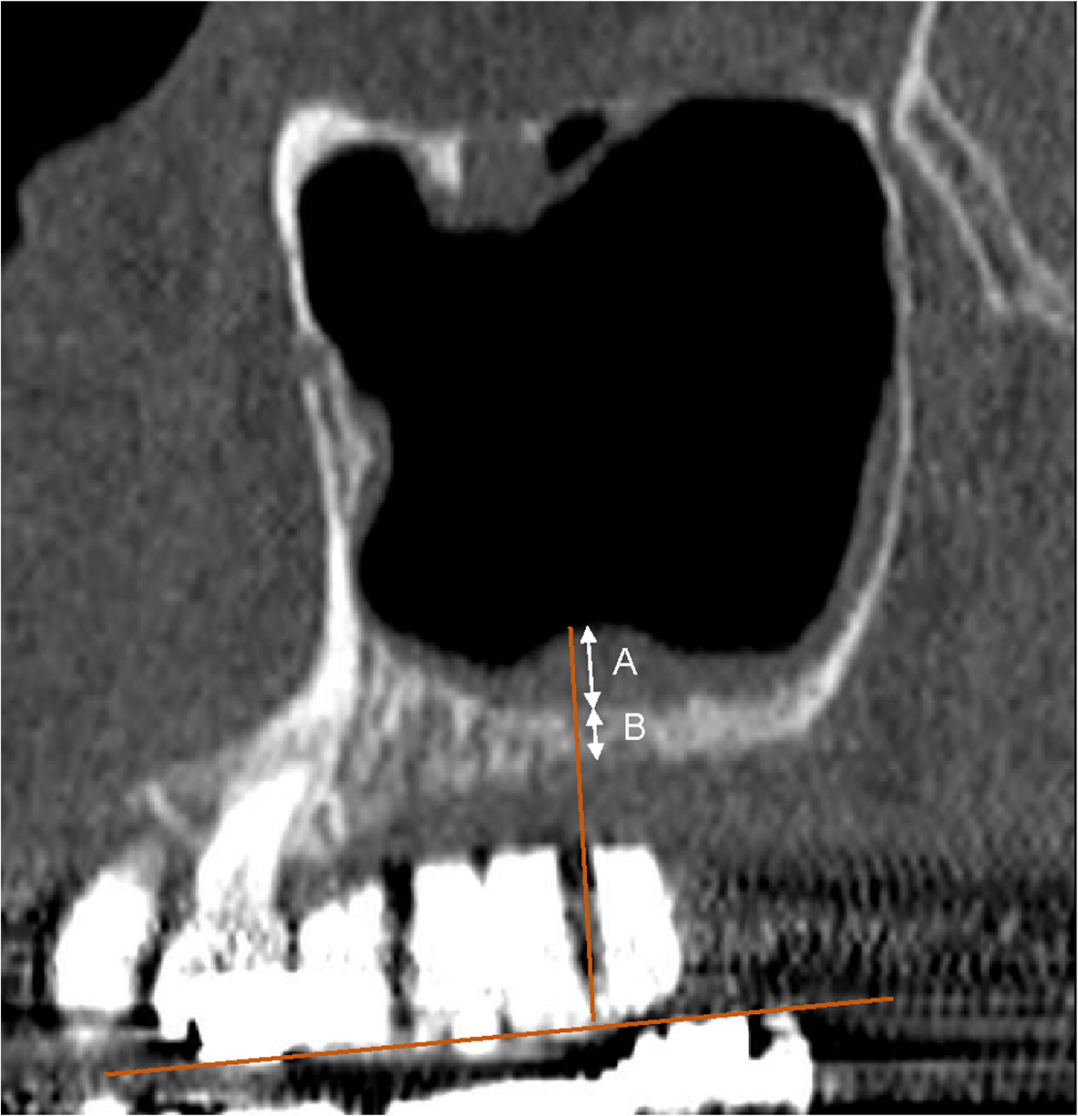
Measurement of Schneiderian mucosal membrane thickness and residual alveolar height perpendicular to the occlusal plane. **a** Maximum mucosal thickness was measured. **b** Minimum residual alveolar bone height was measured

#### Nasal septum deviation measurements (Fig. 2)

Nasal septal deviation (NSD) was defined as any bending of the nasal septal contour observed on coronal CBCT images, in accordance with the definition proposed by Bhandary and Kamath [18]. The patients were divided into three groups according to the measured NSD angles as described by Elahi et al. and Kalabalik et al. [19, 20]: mild (< 9°; group 1), moderate (9°–15°; group 2), and severe (≥ 15°; group 3).

**Fig. 2:**
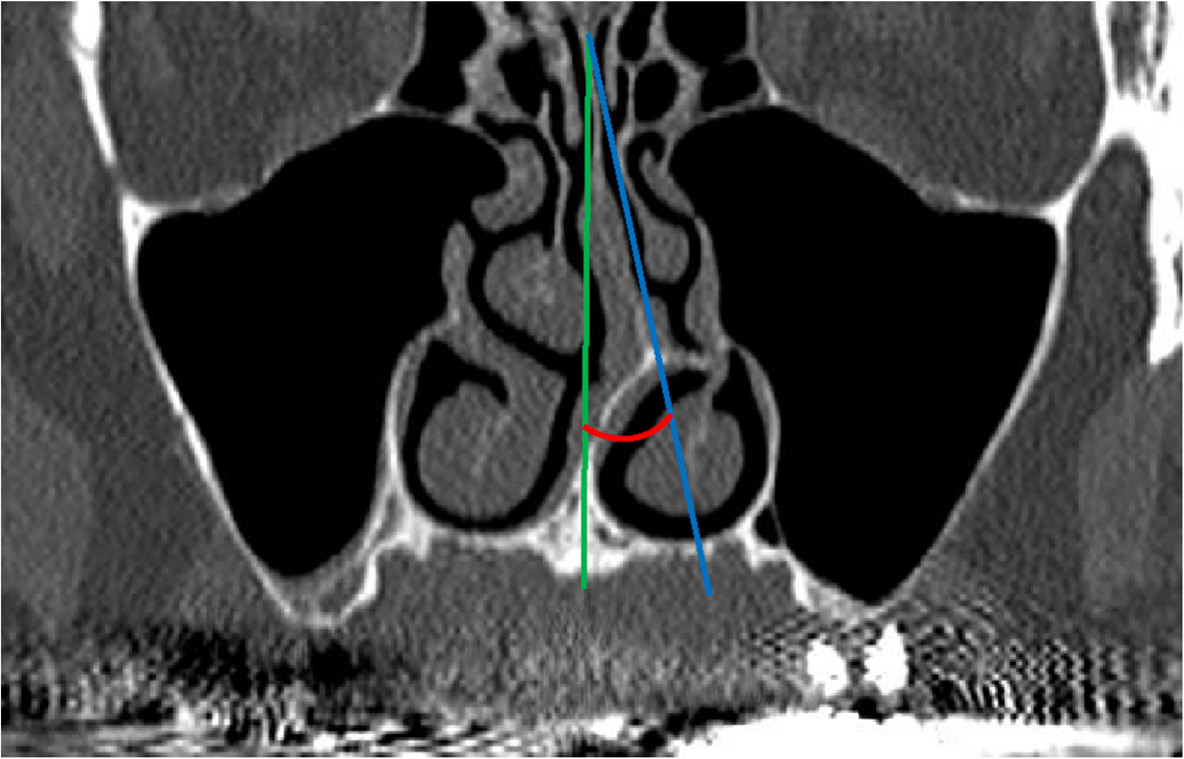
Measurement of nasal septum deviation. The angle between the anterior nasal spine from the crista galli (green line) and the line connecting the most prominent point of the nasal septum from the crista galli (blue line) represents the nasal septum deviation (red)

All imaging measurements based on CBCT were conducted by experienced dental radiology specialists.

### Investigation categories

Relationships between SMT and the following influencing factors were investigated: (1) age, (2) sex, (3) systemic disease, (4) smoking, (5) period after tooth extraction (over half a year or less than half a year), (6) reason for tooth extraction (periapical lesions (PA) or other reason), (7) RBH, (8) sinus septa, and (9) nasal septum deviation. Correlations were also investigated for age and residual bone height.

Furthermore, the odds ratios for the rate of SMT > 2 mm (OR1) or SMT < 0.8 mm (OR2) were calculated in accordance with the report by Testori et al. [7].

### Statistical analysis

Intergroup comparisons were performed using Mann-Whitney *U* test, and correlations were analyzed using Spearman’s correlation, with the significance level set at *p* = 0.05. The odds ratios for SMT > 2 mm and SMT < 0.8 mm were calculated, and investigations were conducted using chi-squared tests (PASW Statistics 18.0; SPSS Inc. SPSS, Japan).

## Results

Clinical characteristics are shown in Table 1. The study population included 35 patients (40 sinuses; 11 male, 24 female), and the average patient age was 58.9 ± 9.0 years (male, 57.9 ± 7.7 years; female, 59.9 ± 9.4 years; age range, 41–79 years). The average SMT was 1.09 ± 1.30 mm; the incidence of SMT > 2 mm was 25.0%; the incidence of SMT < 0.8 mm was 50.0%; and the average RBH was 2.14 ± 1.02 mm. Of the 31 patients with NSD, zero patients were classified in the severe group, 23 were in the mild group, and 12 were in the moderate group (32.2%). The relationships of SMT with individual factors are shown in Tables 2, 3 and 4.

**Table 1:** Clinical characteristics of patients

Parameter	Number (35 patients; 40 sinuses)
Age (years)	58.8 ± 9.4
Male	11
Female	24
Systemic diseases	15
Smoking	8 (male, 7; female, 1)
Septa	4
Nasal deviation	
Mild	23
Moderate	12
Reason for extraction	
Periodontitis	19
Periapical lesions	14
Miscellaneous	7

**Table 2:** Factors influencing sinus mucosal thickness

Variable	Category (mm)	*P* value
Sex	Male: 1.80 ± 1.60	0.0078**
	Female: 0.76 ± 0.92	
Age	< 55 years: 0.78 ± 1.08	0.119
	≥ 55 years: 1.29 ± 1.36	
Systemic diseases	+: 1.37 ± 1.34	0.24
	−: 1.02 ± 1.26	
Time after tooth extraction	< 6 months: 1.76 ± 1.47	0.0075**
	≥ 6 months: 0.74 ± 1.07	
Smoking	+: 1.67 ± 1.58	0.067
	−:0.93 ± 1.11	
Reason for tooth extraction	per (+): 1.66 ± 1.64	0.020*
	per (−): 0.79 ± 0.98	
Residual bone height	< 2.5 mm: 0.94 ± 1.24	0.117
	≥ 2.5 mm: 1.50 ± 1.33	
Septa	+: 2.39 ± 1.80	0.0076**
	−:0.91 ± 1.13	
Nasal division	+: 1.65 ± 1.58	0.038*
	−:0.86 ± 1.20	

*p < 0.05; **p < 0.01

**Table 3:** Factors influencing sinus mucosal thickness (> 2 mm rate: odds ratio)

Variable	Category (> 2 mm rate)	OR1	*P* value
Sex	Male: 46.2%	6.86	0.0035**
	Female: 11.1%		
Age	< 55 years: 22%	1.26	0.57
	≥ 55 years: 28%		
Systemic diseases	+: 33.3%	2.60	0.51
	−: 19.4%		
Time after tooth extraction	< 6 months: 35.7%	2.33	0.25
	≥ 6 months: 19.2%		
Smoking	+: 44.4%	4.21	0.13
	−: 16.1%		
Reason for tooth extraction	per (+): 50.0%	7.67	0.0073**
	per (−): 11.5%		
Residual bone height	< 2.5 mm: 20.7%	3.84	0.31
	≥ 2.5 mm: 36.3%		
Septa	+: 60.0%	6.00	0.22
	−: 20.0%		
Nasal division	+: 41.6%	3.29	0.11
	−: 17.9%		

*p < 0.05; **p < 0.01

**Table 4:** Factors influencing sinus mucosal thickness (< 0.8 mm rate: odds ratio)

Variable	Category (< 0.8 mm rate)	OR2	*P* value
Sex	Male: 38.5%	2.00	0.31
	Female: 55.6%		
Age	< 55 years: 66.7%	3.00	0.10
	≥ 55 years: 40.0%		
Systemic diseases	+: 33.3%	2.43	0.26
	−: 54.8%		
Time after tooth extraction	< 6 months: 21.4%	6.93	0.008**
	≥ 6 months: 65.4%		
Smoking	+: 44.4%	1.33	0.70
	−: 51.6%		
Reason for tooth extraction	per (+): 42.9%	1.56	0.51
	per (−): 53.8%		
Residual bone height	> 2.5 mm: 55.1%	2.15	0.28
	≥ 2.5 mm: 36.3%		
Septa	+: 25.0%	3.39	0.29
	−: 52.8%		
Nasal division	+: 25.0%	4.64	0.038*
	−: 60.7%		

*p < 0.05; **p < 0.01

### Sex

The average SMTs in males and females were 1.80 ± 1.60 mm and 0.76 ± 0.92 mm, respectively, with males showing significantly higher values (*p* = 0.0078). The rates of SMT > 2 mm in males and females were 46.2% and 11.1%, respectively, with the proportion being significantly higher in males (*p* = 0.0035, OR1: males, 6.86). The rates of SMT < 0.8 mm in males and females were 38.5% and 55.6%, respectively, with no significant difference observed, although the rate in females tended to be higher (*p* = 0.31, OR2: females, 2.00).

### Age

Analyses based on age showed that SMT tended to increase with age, but no correlations were observed (*r* = 0.087, *p* = 0.296). The average SMT in those over and under 55 years of age were 1.29 ± 1.36 mm and 0.78 ± 1.08 mm, respectively, and the difference was not significant. The rates of SMT > 2 mm in those over and under 55 years of age were 28% and 20%, respectively, with virtually no difference between them (*p* = 0.57, OR1 1.26). The rates of SMT < 0.8 mm in those over and under the age of 55 years were 40.0% and 66.7%, respectively, with the OR being higher in those under 55 years of age, although no significant difference was observed (*p* = 0.10, OR2 3.00).

### Systemic disease

The average SMTs in patients with systemic disease (+) and (−) statuses were 1.37 ± 1.34 mm and 1.02 ± 1.26 mm, respectively, although the difference was not significant (*p* = 0.24). The rates of SMT > 2 mm in those with systemic disease (+) and (−) statuses were 33.3% and 19.4%, respectively, and the OR for SMT > 2 mm was higher in those with systemic disease (+) status, although the difference was not significant (*p* = 0.51, OR1 2.60). The rates of SMT < 0.8 mm in those with systemic disease (+) and (−) statuses were 33.3% and 54.8%, respectively, and the OR for SMT < 0.8 mm was higher in those with systemic disease (−) status, although the difference was not significant (*p* = 0.26, OR2 2.43).

### Period after tooth extraction

The average SMTs for periods less than half a year and more than half a year were 1.76 ± 1.47 mm and 0.74 ± 1.07 mm, respectively, and the difference was statistically significant (p = 0.0075). The rates of SMT > 2 mm for periods less than half a year and more than half a year were 35.7% and 19.2%, respectively, and the OR for SMT > 2 mm was higher for periods less than half a year, although no significant difference was observed (p = 0.25, OR1 2.33). The rates of SMT < 0.8 mm for periods less than half a year and more than half a year were 21.4% and 65.4%, respectively, and the OR for SMT < 0.8 mm was higher for periods more than half a year, although no significant difference was observed (p = 0.008, OR2 6.93).

### Smoking

The average SMTs for smoking (+) and (−) statuses were 1.67 ± 1.58 mm and 0.93 ± 1.11 mm, respectively, although the difference was not significant (*p* = 0.067). The rates of SMT > 2 mm for smoking (+) and (−) statuses were 44.4% and 16.1%, respectively, and the smoking (+) status showed a higher OR, although no significant difference was observed (*p* = 0.13; OR1 4.21). The rates of SMT < 0.8 mm for smoking (+) and (−) statuses were 44.4% and 51.6%, respectively, with no significant difference between them (*p* = 0.70; OR2 1.33).

### Reason for tooth extraction

The average SMTs for PA (+) and (−) cases were 1.66 ± 1.64 mm and 0.79 ± 0.98 mm, respectively, and PA in the tooth-extracted area resulted in significantly larger SMT (*p* = 0.020). The rates of SMT > 2 mm for PA (+) and (−) cases were 50.0% and 11.5%, respectively, and the difference between the groups was significant (*p* = 0.0073, OR1 7.67). Meanwhile, the rates of SMT < 0.8 mm for PA (+) and (−) cases were 42.9% and 53.8%, respectively, and the difference was not significant (*p* = 0.51; OR2 1.56).

### Residual bone height

In assessments of RBH, SMT tended to increase with increased bone height, but no correlations were observed (*r* = 0.057, *p* = 0.363). The average SMTs for RBHs above and below 2.5 mm were 1.50 ± 1.33 mm and 0.94 ± 1.24 mm, respectively; SMT increased with RBH, but no significant difference was observed (*p* = 0.117). The rates of SMT > 2 mm for RBH above and below 2.5 mm were 36.3% and 20.7%, respectively, and the difference was not significant (*p* = 0.31, OR1 3.84). The rates of SMT < 0.8 mm for RBH above and below 2.5 mm were 36.3% and 55.1%, respectively, and the difference was not significant (*p* = 0.28, OR2 2.15).

### Sinus septa

The average SMTs for septa (+) and (−) statuses were 2.39 ± 1.80 mm and 0.91 ± 1.13 mm, respectively, with the septa (+) status showing a significantly higher SMT (*p* = 0.0076). The rates of SMT > 2 mm for septa (+) and (−) statuses were 60.0% and 20.0%, respectively, although the difference was not significant (*p* = 0.22, OR1 6.00). The rates of SMT < 0.8 mm for septa (+) and (−) statuses were 25.0% and 52.8%, respectively, with septa (−) showing a higher OR of the rate of SMT < 0.8 mm, although the difference was not significant (*p* = 0.29, OR2 3.39).

### Nasal septum deviation

The average SMTs for NSD (+) and (−) statuses were 1.65 ± 1.58 mm and 0.86 ± 1.20 mm, respectively, with the NSD (+) status showing a significantly higher SMT result (*p* = 0.038). The rates of SMT > 2 mm for NSD (+) and (−) statuses were 41.6% and 17.9%, respectively, with NSD (+) status showing a higher OR for SMT > 2 mm, although the difference was not significant (*p* = 0.11, OR1 3.29). The rates of SMT < 0.8 mm for NSD (+) and (−) statuses were 25.0% and 60.7%, respectively, with the NSD (−) status showing a significantly higher rate of SMT < 0.8 mm (*p* = 0.038; OR2 4.64).

## Discussion

In maxillary sinusitis accompanying implant treatment, local inflammation accompanying maxillary sinus floor augmentation procedures may result in interactions among infection, caused by decreased mucociliary functions that conduct maxillary sinus ventilation/discharge, as well as microbes, bacteria, and viruses; and ostium/ostiomeatal complex occlusion due to nasal/paranasal sinus morphology, all of which result in the onset of acute sinusitis [21].

The average SMT in the present study was 1.09 ± 1.30 mm, the rate of SMT > 2 mm was 25.0%, and the rate of SMT < 0.8 mm was 50.0%. Zimmo et al. [22] measured the SMT below teeth using CBCT and reported that the average SMT was 1.81 ± 1.66 mm, and the rate of SMT > 2 mm was 29.2%. Meanwhile, systematic reviews by Monje et al. [23] on edentulous areas following tooth extraction showed that the average SMT in edentulous areas measured by MDCT and CBCT was 1.33 ± 1.7 mm, and the rate of SMT > 2 mm in the edentulous area was 40.1%. CBCT-based investigations by Shanbhag et al. reported that the rate of SMT > 2 mm was 53.6% [24]. Nunes et al. reported that the prevalence of SMT > 2 mm in the edentulous area was 34.9%, and the prevalence of SMT < 1 mm was 39.3% [25]. Furthermore, Dursun et al. [26] conducted investigations on SMT based on the presence of teeth and showed that tooth loss resulted in significantly higher SMT. The present research results reported thinner results than those presented by Monje et al. and Shanbhag et al., and the reason for this was thought to be due to racial differences, since our study only investigated Japanese populations. With respect to the relationship between sinus mucosal perforation and SMT, Al-Dajani [27] conducted a meta-analysis on risk factors for sinus mucosal perforation and reported that the risk of sinus mucosal perforation increased with a smaller SMT. Furthermore, Lum et al. [28] reported that perforated and non-perforated SMTs were 0.84 ± 0.67 mm and 2.65 ± 4.02 mm, respectively, and that increased SMTs resulted in a significantly decreased risk of sinus mucosal perforation. Kezys et al. [29] conducted a systematic review in which the risk of sinus mucosal perforation was reported to be the lowest when SMT was between 1 and 2 mm. Thus, the fact that sinus mucosal perforation is more likely to occur during maxillary sinus floor augmentation in Asian populations such as the Japanese needs to be sufficiently considered, based on the present research results.

The present study showed that sex, time after tooth extraction, reason for tooth extraction, sinus septa, and nasal septum deviation were influential factors.

With respect to sex, Dursun et al. [26] conducted investigations on SMT due to the presence of teeth and showed that SMT was significantly higher in males compared to females. Ramanauskaite et al. [30] also conducted investigations on SMT in the edentulous area and various local factors and showed that local factors like residual bone height and bone absorption were not correlated, but that males had a significantly higher thickness. Furthermore, Aksoy et al. [31] conducted a similar investigation and showed that the rates of SMT > 2 mm in males and females were 52.8% and 35.9%, respectively, with males showing significantly higher thickness. Similar to the findings reported by Ramanauskaite et al., Aksoy et al., and Durson et al., our research also showed that males had significantly greater thickness. As for why males showed greater thickness, a systematic review by Monje et al. relating to SMT reported that patient-related factors showed no significant differences, but that smoking, age, and periodontal diseases influenced SMT, and smoking in particular showed significantly thicker results, with SMT in smokers and non-smokers being 2.64 mm and 1.05 mm, respectively. The present research also showed increasing tendencies of SMT in smokers (*p* = 0.067), and a higher percentage of smokers who were male (7 out of 9 sinuses in our research).

With respect to the time after tooth extraction, Tassoker [32] conducted investigations into the risk factors for maxillary sinus pathology with CBCT and showed minimal correlations with sinus septa, concha bullosa, and nasal septal deviation, and noted that periapical lesions were a major risk factor that increased maxillary sinus pathology. Among assessments of changes after tooth extraction, Block et al. [33] conducted research that compared changes in the sinus mucosa before and after tooth extraction in patients planning to undergo implant treatment and showed that SMT decreased but did not completely disappear in CBCT images taken 3–6 months after tooth extraction for areas where SMT was > 2 mm prior to tooth extraction. Yoo et al. [34] conducted 3-group investigations on differences in SMT based on time after tooth extraction and showed that the average SMT less than 4 months after tooth extraction was 3.95 ± 3.45 mm, 2.58 ± 2.10 mm for 4–11 months, and 1.87 ± 1.62 mm for over 12 months, with SMT significantly decreasing as the time after tooth extraction becoming longer.

Among assessments of SMT and the reasons for tooth extraction, Janner et al. [13] conducted investigations on SMT and dental conditions and showed that the influence of periodontal status was significantly higher than that of the periapical status. Reports on the relationship between periapical lesions and SMT in the maxillary molar area by Sakir and Yalcinkaya [35] showed that SMT changed based on the size of periapical lesions in teeth adjacent to the maxillary sinus. Shanbhag et al. [36] also conducted investigations on the relationship between periapical lesions and SMT and showed that this had a strong correlation with SMT > 2 mm, with odds ratio = 9.75, and *p* < 0.001. Furthermore, a systematic review by Eggmann et al. [37] showed that on CBCT scans, periapical lesions in the posterior maxilla were likely to be associated with Schneiderian membrane thickening. In contrast, current evidence regarding the relationship between periodontal diseases and the appearance of the Schneiderian membrane in CBCT was inconclusive. The research design is different as the present study is a novel study related to the reasons for tooth extraction in the edentulous area, but our results were similar to those of research with teeth present.

Various reports have indicated the prevalence of sinus septa from 16 to 35.4%. The prevalence of sinus septa in the edentulous area had been reported as 26.59% [38, 39]. A meta-analysis by Al-Dajani [27] indicated that a thin membrane and sinus septa were risk factors that increased sinus membrane perforation, but there are very few reports on the relationship between SMT and sinus septa. Sanchez-Perez et al. [40] and Cakur et al. [41] reported that septa presence was negatively correlated with SMT. However, Rancitelli et al. [38] reported that SMT in areas without a septa and adjacent to the septa were 0.85 mm and 1.8 mm, respectively, with the latter showing significantly thicker results; 17.5% and 23.4% of patients had thickening > 6 mm in the absence of septa group and presence of septa group, respectively, with the latter showing increased results.

With respect to the relationship between SMT and nasal septum deviation, the prevalence of nasal septum deviation has been shown to be between 26.6% and 58%, and there are various measurement methods and classifications [42]. Bayrak et al. [43] reported that there was no correlation between nasal septum deviation and SMT. Meanwhile, Taghiloo and Halimi [44] investigated changes in SMT based on differences in nasal septum deviation type and showed that there was no difference based on nasal septum deviation type, but increased SMT was observed in 31.76% and 56.67% of males and females with nasal septum deviation, respectively, and there was a significant relationship between nasal septum deviation and thickening of maxillary sinus mucosa. Furthermore, research based on CBCT by Shanbhag et al. [24] showed that ostium obstruction was observed in 13.1% of patients, and that there was a correlation with SMT. With respect to the relationships with the ostiomeatal complex, further detailed investigations are necessary due to the issues related to the definition of nasal septum deviation and measurement methods, as well as the influence of the thickness of the middle nasal concha, ostium size, concavity/convexity of the nasal septum deviation, and medical history of chronic sinusitis.

As such, a treatment plan that sufficiently considers anatomical factors including OMC, sex, smoking, RBH, patient-related factors like sinus septa, reason for tooth extraction, time after tooth extraction, and dental factors like the extent of tooth root protrusion in adjacent teeth is required in order to safely conduct maxillary sinus floor augmentation.

However, as this study was limited in terms of using only CBCT investigations before the maxillary sinus floor augmentation surgery, the direct relationship between the surgery and intraoperative and postoperative complications has not been investigated. Furthermore, we think that the risk factors associated with the maxillary sinus augmentation and implantation procedure are more important than those associated with preoperative SMT, in terms of preventing intraoperative and postoperative complications.

In the future, we will investigate the relationship between perforation of the sinus mucosa and postoperative infection, and examine the risk factors for maxillary sinus floor augmentation.

## Conclusions

The following conclusions were obtained with respect to factors that influence SMT:

1. Factors that increase SMT include sex (male), time after tooth extraction < 6 months, periapical lesions, sinus septa, and nasal septum deviation.
2. Factors related to SMT > 2 mm include sex and reason for tooth extraction, while factors relating to SMT < 0.8 mm were time after tooth extraction and nasal septum deviation.

The present research is limited, so further detailed research is needed.

## Data Availability

The datasets of the current study are available from the corresponding author on reasonable request.

## Abbreviations

CBCT: Cone-beam computed tomography
CT: Computed tomography
MDCT: Multidetector computed tomography
NSD: Nasal septum deviation
OMC: Ostiomeatal complex
PA: Periapical lesion
RBH: Residual alveolar bone height
SMT: Sinus mucosal thickness
